# Supplementary material for: Utilizing Protein–Peptide Hybrid Microarray for Time-Resolved Diagnosis and Prognosis of COVID-19
Source: Microorganisms. 2023 Sep 28;11(10):2436. doi: 10.3390/microorganisms11102436 (PMC10609375; doi:10.3390/microorganisms11102436)
Supplement: Supplementary file 1 [file microorganisms-11-02436-s001.zip › microorganisms-2555715-SI.pdf]

Supplementary Materials for

Utilizing Protein–Peptide Hybrid Microarray for Time-Resolved Diagnosis and Prognosis of COVID-19

Peiyan Zheng [1,†], Baolin Liao [2,†], Jiao Yang [3], Hu Cheng [3,4], Zhangkai J. Cheng [1], Huimin Huang [1], Wenting Luo [1], Yiyue Sun [3,4], Qiang Zhu [5], Yi Deng [3,4], Lan Yang [3], Yuxi Zhou [3], Wenya Wu [3,4], Shanhui Wu [1], Weiping Cai [2], Yueping Li [2], Xiaoneng Mo [2], Xinghua Tan [2], Linghua Li [2], Hongwei Ma [3,*] and Baoqing Sun [1,*]

1 Department of Clinical Laboratory, National Center for Respiratory Medicine, National Clinical Research Center for Respiratory Disease, State Key Laboratory of Respiratory Disease, Guangzhou Institute of Respiratory Health, The First Affiliated Hospital of Guangzhou Medical University, Guangzhou 510120, China
[2] Guangzhou Institute of Clinical Medicine of Infectious Diseases, Guangzhou Eighth People's Hospital, Guangzhou Medical University, Guangzhou 510440, China
[3] Division of Nanobiomedicine, Suzhou Institute of Nano-Tech and Nano-Bionics, Chinese Academy of Sciences, Suzhou 215123, China
[4] Nano Science and Technology Institute, University of Science and Technology of China, Suzhou 215123, China
[5] State Key Laboratory of Respiratory Disease, Guangzhou Institutes of Biomedicine and Health Chinese Academy of Sciences, Guangzhou 510530, China
***** Correspondence: hwma2008@sinano.ac.cn (H.M.); sunbaoqing@vip.163.com (B.S.)
[†] These authors contributed equally to this work.

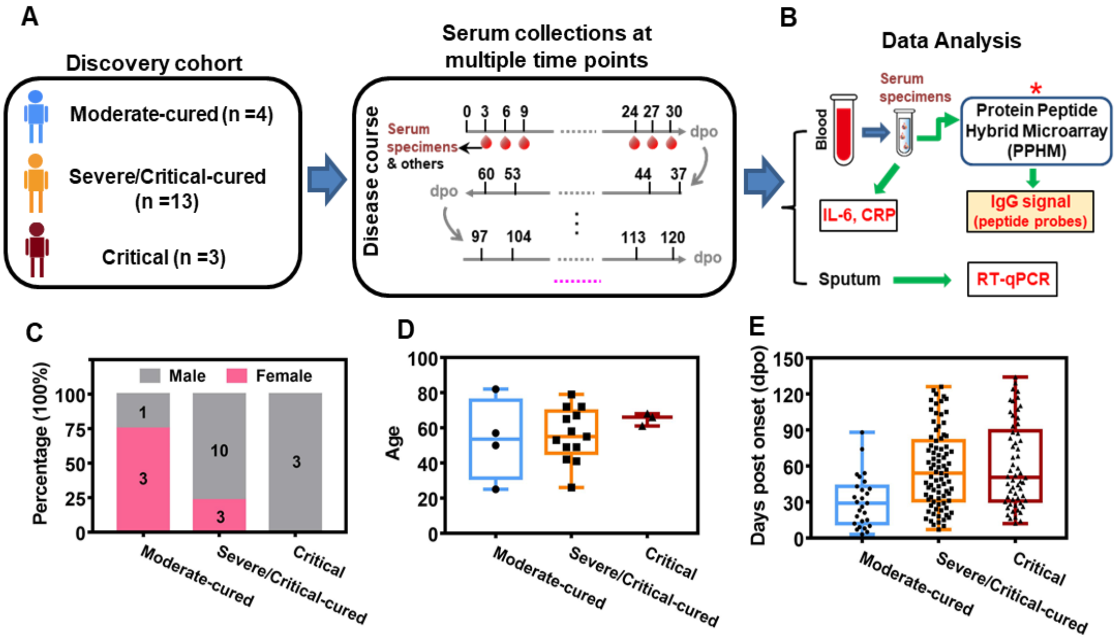

Figure S1. Study strategy and basic information about the COVID-19 patients. (**A**) Three groups of COVID-19 patients were identified in the discovery cohort (left), for which we had a total of 323 serum samples and other samples (e.g., ~ 500 sputum samples) collected throughout the disease course of patients, the serum samples were sampled as indicated (right). Briefly, the collection of serum sample was performed starting from patient admission to the hospital and continued until the days close to patient discharge. The time interval for serum collections was 3~4 days for the first 30 days, and about once a week afterwards. Note that the disease course for some of the patients in the critical group lasted up to ~120 days or even longer since the days post onset (d.p.o.). (**B**) A Part of the collected serum samples were assessed for examining IL-6 and CRP levels. All of the serum samples were monitored for the presence of antibodies against whole protein and/or peptide probes derived from SARS-CoV-2 proteins using a protein peptide hybrid microarray (PPHM). For sputum samples, quantitative reverse transcription PCR (RT-qPCR) test was performed for the diagnosis of SARS-CoV-2. (**C**) The gender distribution for the identified three groups of COVID-19 patients. (**D**) The age distribution for each group. The center line in each box represents the median age. (**E**) The number of days between symptom onset and the serum sample collections at different time points for three different patient groups from the discovery cohort. Each data point indicates the d.p.o. of a single patient at each time point. The center line in each box shows the median d.p.o.; the bottom and top lines represent the minimal and maximal d.p.o. in each patient group, respectively.

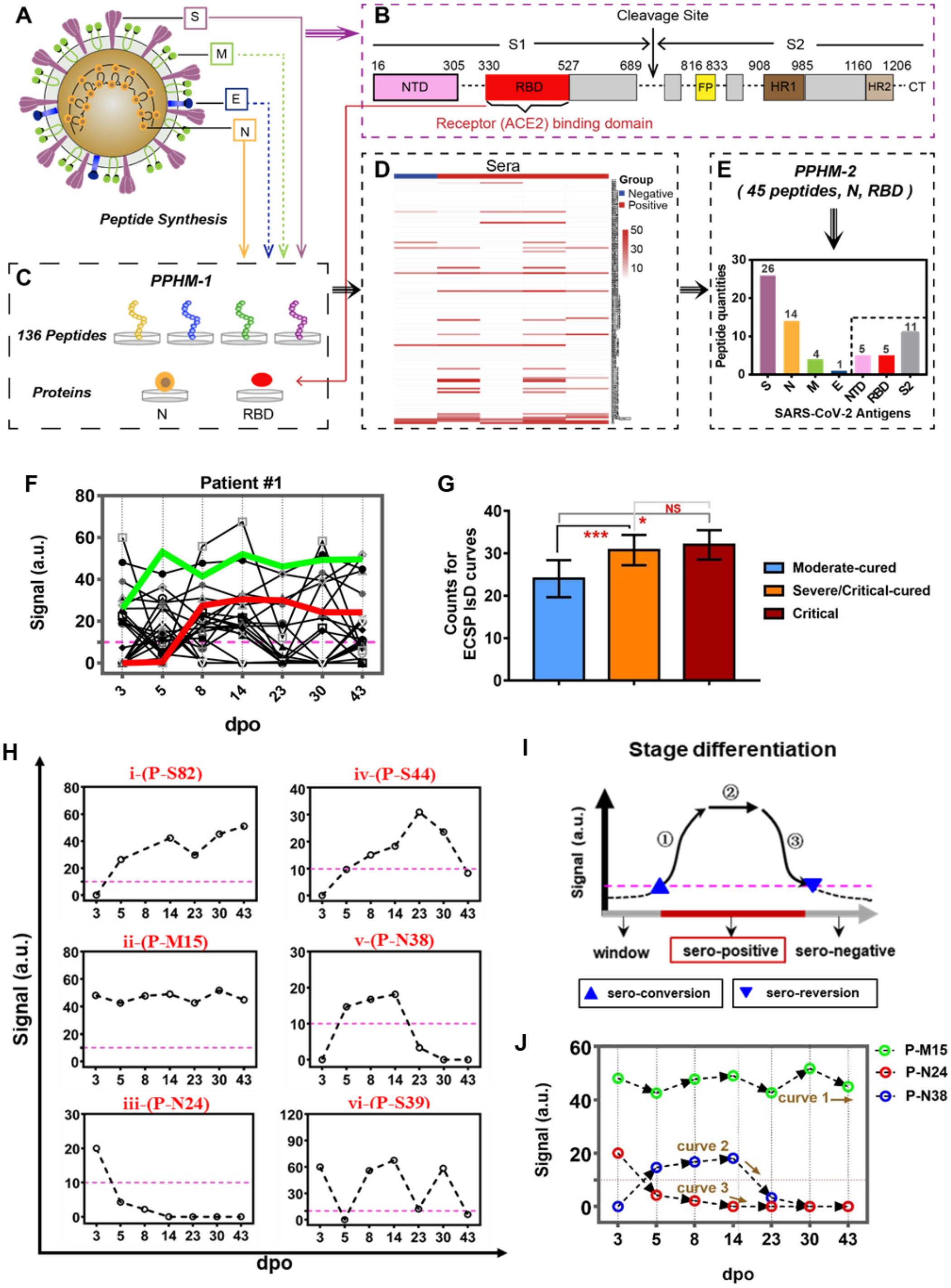

Figure S2. Multiple phases of sero-antibody dynamics (MPAD) are evident in PPHM screening results. (**A-E**) Two-level PPHM screening process. (**A**) The structural proteins of SARS-CoV-2. (**B**) Schematic of the SARS-CoV-2 S protein structure, emphasizing the RBD (receptor binding domain, red). (**C**) PPHM-1 comprises the screening probes of 136 peptides and two proteins (N and RBD) for the first level

screening. (**D**) A heat map representing the first level screening of five serum samples: one negative sample from a healthy donor (blue region on top left), and four samples from COVID-19 patients (red region on top). (**E**) PPHM-2 comprises 45 peptides which were identified as the potential "epitope containing short peptides" (ECSPs) of SARS-CoV-2 through the first level screening; these peptides were used for the second level screening to detect the IgG levels of a large longitudinal serum samples from the discovery cohort over time. The quantified levels of potential ECSPs (originating from each viral protein) for the detection of SARS-CoV-2 Linear B cell epitopes are displayed with colored columns. (**F**) The protein and ECSP "IgG sero dynamic" (IsD) curves in Patient #1. The protein IsD curves of the N protein (green curve), RBD (red curve); and 22 ECSP IsD curves of ECSPs originating from four structural proteins of SARS-CoV-2 (black curves). The signal threshold is presented as a pink dashed line. (**G**) Comparison of the counts for the ECSP IsD curves in three different patient groups. Student's t-tests were used to assess differences among groups; * indicates $p < 0.05$; ** indicates $p < 0.01$; *** indicates $p < 0.001$. (**H**) Representative six different types of ECSP IsD curves for Patient #1: (**H-i**) a rising curve; (H-ii) an almost straight line; (**H-iii**) a declining curve; (**H-iv**), (**H-v**), and (**H-vi**) are ECSP IsD curves representative of more than one changing trend, i.e., combinations with rising curves, straight lines, and/or declining curves. (**I**)The three stages of the antibody life cycle (Long et al., 2020). (**J**) Miniature "multiple phases of sero-antibody dynamics" (MPAD) mining in the IsD curves of Patient #1. Three different ECSP IsD curves were randomly selected from Fig. 2H.

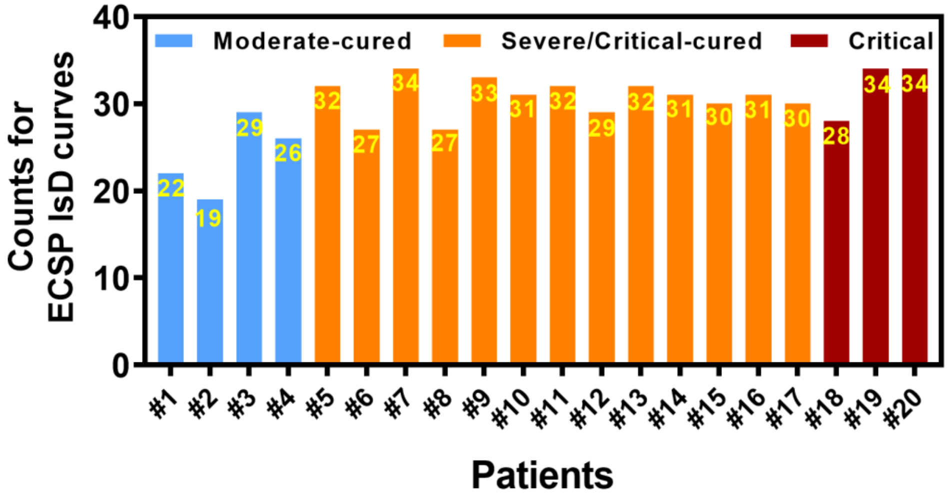

Figure S3. The counts for the ECSP IsD curves in ecah COVID-19 patient from the discovery cohort. The ECSP IsD curves for each patient were cunted based on the PPHM-2 screening results. The quantity for the counts of the ECSP IsD curves in each patient was displayed on the top of each column.

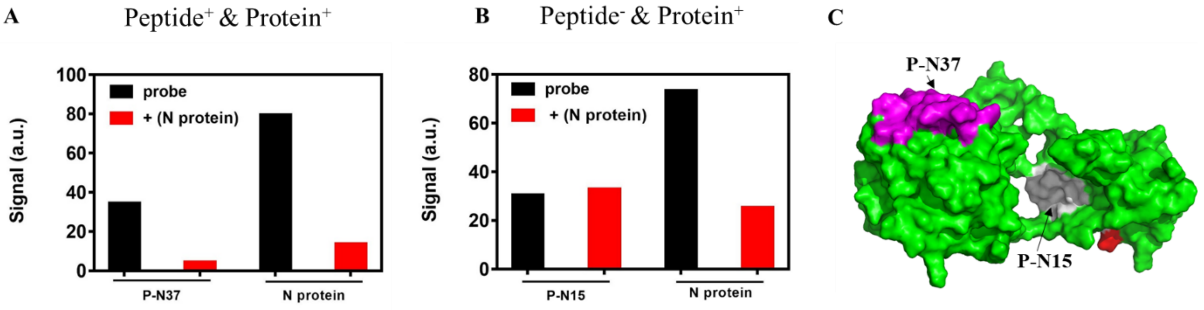

Figure S4. N protein blocking experiment. Free N protein was individually doped to (**A**) the serum specimen of Patient #10 at 70 dpo and (**B**) the serum specimen of Patient #15 at 63 dpo before screening against PPHM-2. **(A)** Both anti-(P-N37) IgG and anti-(N protein) IgG were blocked by free N protein; **(B)** N protein can not block the signal values of anti-(P-N15) IgG, but can block the signal values of anti-(N protein) IgG. **(C)** The surface presentation of SARS-CoV-2 N protein (https://zhanglab.ccmb.med.umich.edu/COVID-19/, QHD43423.pdb). P-N37 (purple) is exposed at the protein surface and P-N15 (grey part) is majorly buried inside of the protein.

A

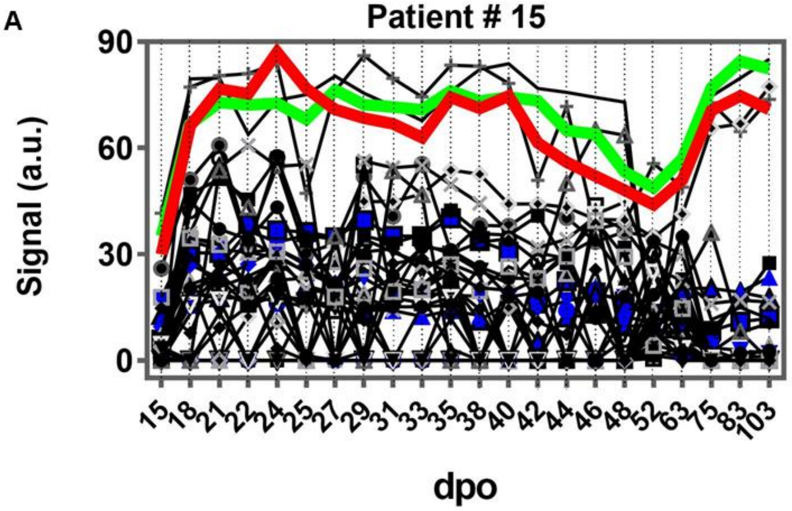

B

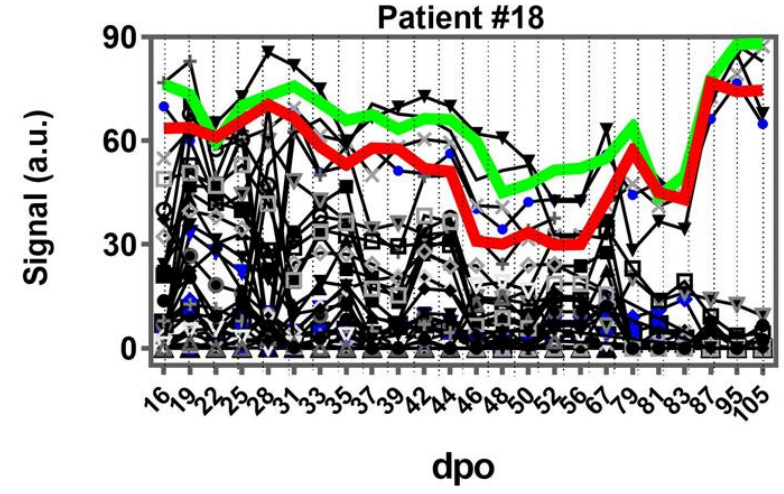

Figure S5. The "multiple phases of antibody sero-dynamics" (MPAD) in two representative COVID-19 patients. (A) The MPAD (within ~ 100 dpo) in Patient #15 who is from Severe/Critical-cured patient group. **(B)** The MPAD (within ~ 100 dpo) in Patient # 18 who is from the Critical patient group. The MPAD comprises the protein IsD curves of the N protein (green), RBD protein (red) and the ECSP IsD curves (black).

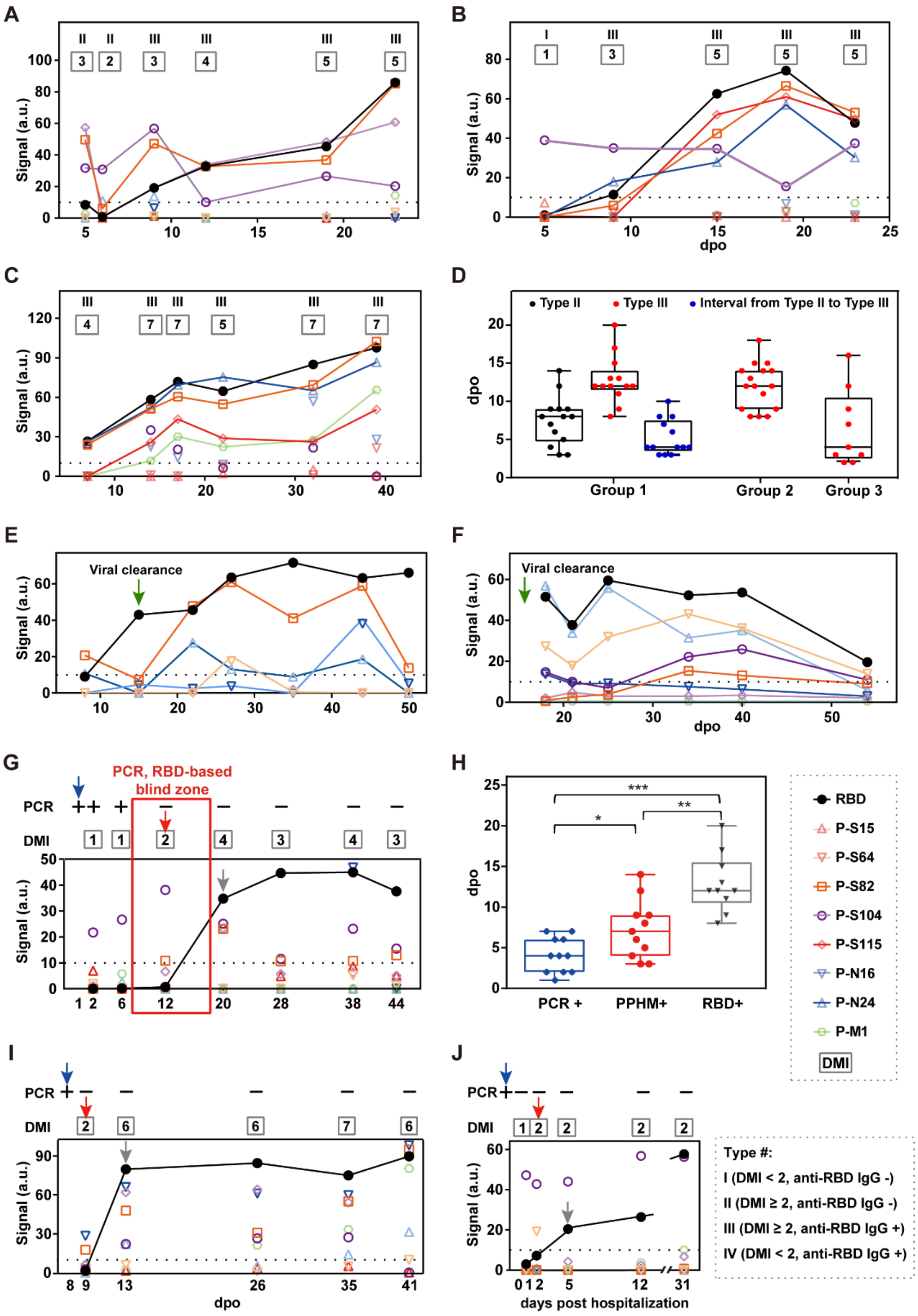

Figure S6. Some anti-ECSP IgGs showed earlier sero-conversion than anti-RBD IgG and some anti-ECSP IgGs are short lived compared with anti-RBD IgGs. (A) According to appearing sequence of the four type results of PPHM COVID-19 assay, the quarantine cohort can be divided into three groups. Patient #E7 representing Group 1 showed Type #2 and Type #3 results. **(B)** Patient #E15 representing

Group 2 showed Type #1 and Type #3 results. **(C)** Patient #E36 representing Group 3 showed only Type #3 results. **(D)** The distribution of three d.p.o.: i) when first appearance of Type #2, ii) Type #3 results, and iii) the difference between i and ii for each patient. **(E)** The group as represented by Patient #E12 showed some anti-ECSP IgGs are short lived compared with anti-RBD IgGs. **(F)** Patient #3 showed anti-ECSP IgGs and anti-RBD IgGs were nearly eliminated or obviously declining within the observation period after viral clearance. The red arrow indicating viral absence. **(G)** Symptomatic patient #E5 sequentially showed positive results for PCR, PPHMCOVID-19, and RBD-based serological assays, revealing a "blind zone" existed in this patient's clinical data for an 8-day period if using PCR and RBD-based serological assays. **(H)** Distribution of the d.p.o.s of the first positive results for three assays in 11 symptomatic patients who had at least two PCR positive results. Representative cases of total 25 patients who showed only one PCR positive result, **(I)** symptomatic patient #E1 and (J) asymptomatic patient #E44, indicating the end of detecting zone of PCR test.

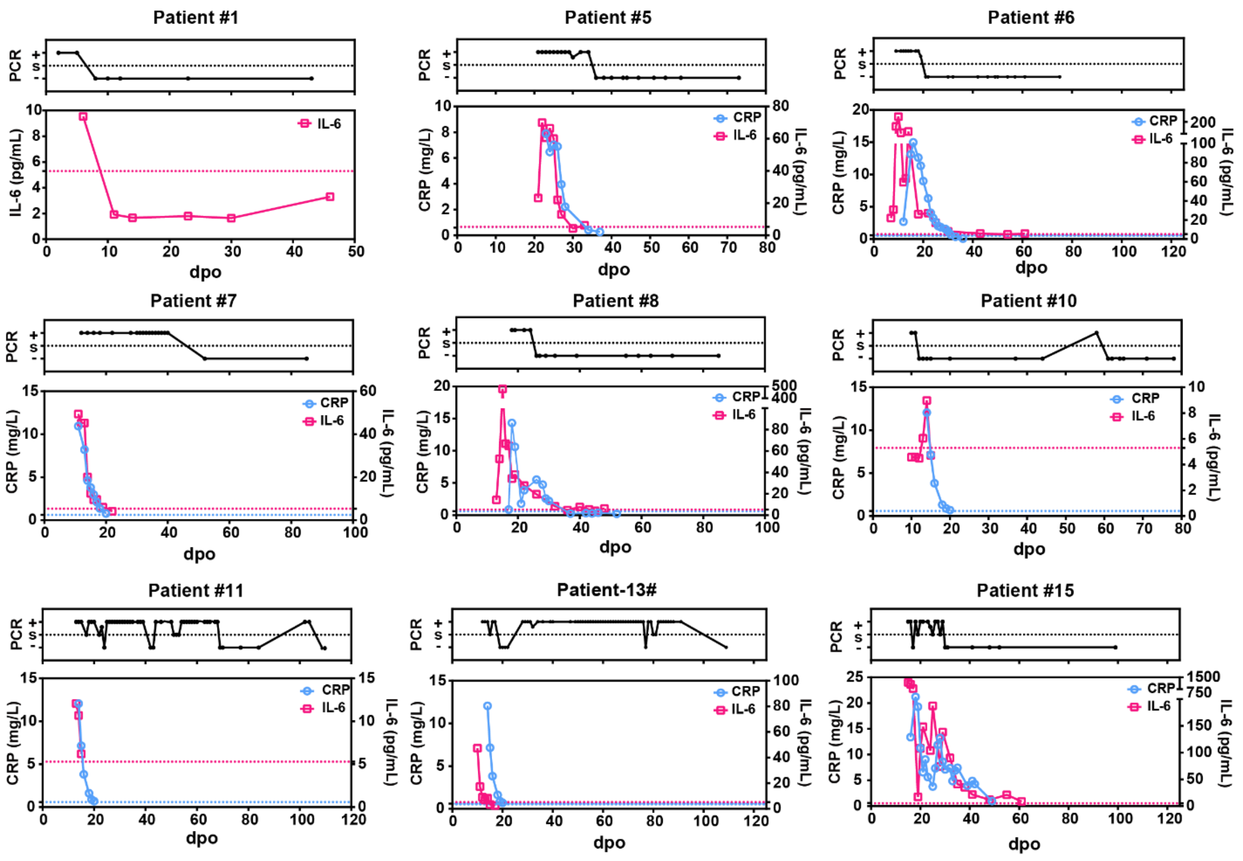

Figure S7. The dynamic curves of CRP and IL-6 in individual patient. We tracked the dynamic changes of infection-related biomarkers IL-6 (mauve) and CRP (blue) in each patient. The patients presented here were all recovered from the disease. Based on their PCR data, we found that all the PCR results finally turned as negative, which indicated the disease recovery and was consistent with their disease outcomes. The serum levels of IL-6 and/or CRP all dropped (near) to the normal level in these patients, indicating the good prognosis as well. The threshold for normal levels of IL-6 and CRP are presented with dashed lines of the corresponding color.

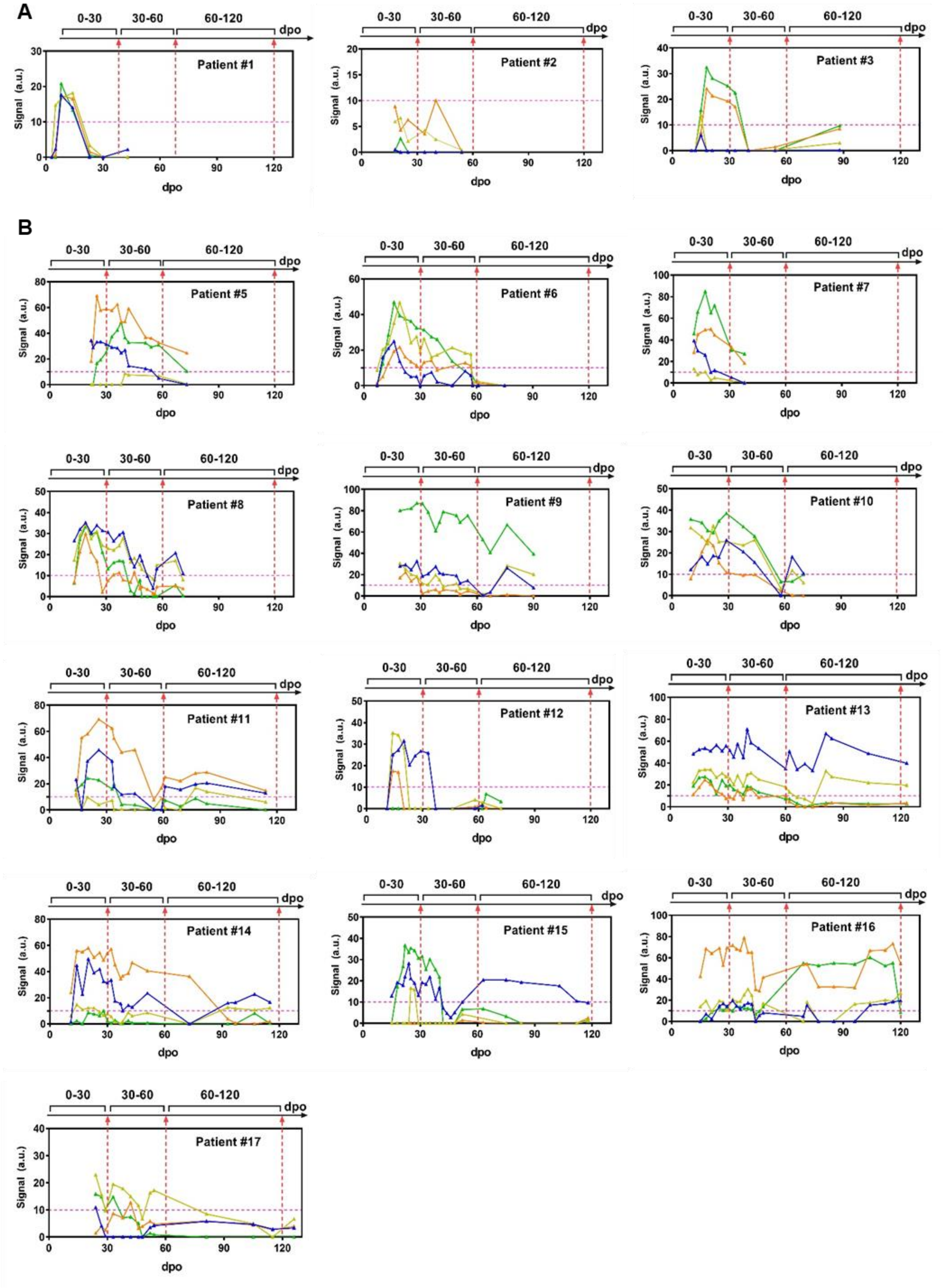
A
B
0-30
30-60
60-120
dpo
Signal (a.u.)
Patient #1
Patient #2
Patient #3
Patient #5
Patient #6
Patient #7
Patient #8
Patient #9
Patient #10
Patient #11
Patient #12
Patient #13
Patient #14
Patient #15
Patient #16
Patient #17

C

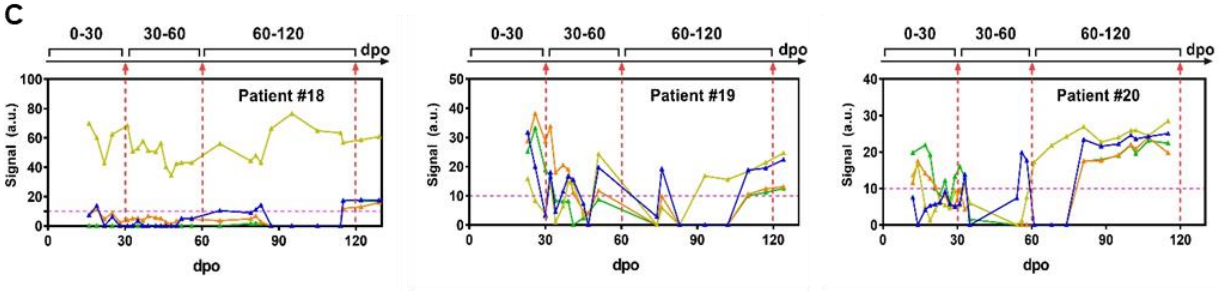

Figure S8. The ECSP IsD curves of P-N37~P-N40 in individual patient. The P-N37~P-N40 IsD curves of 19 COVID-19 patients was individually examined. The characteristics of the ECSP IsD curves of P-N37~P-N40 for each patient were consistent with the previous descriptions of the corresponding patient group, as shown in Fig. 3C and Fig. 3D. A (Moderate-cured group); B (Severe/Critical-cured group); C (Critical group).

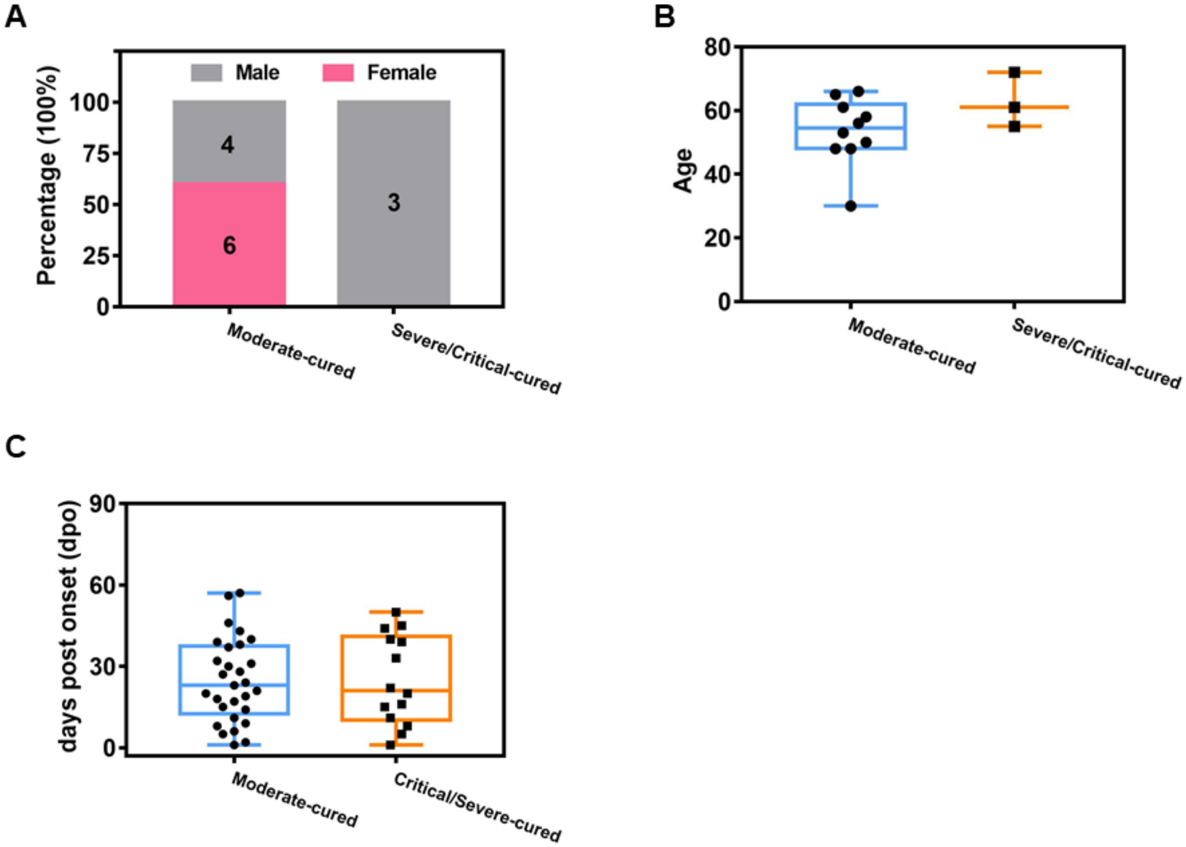

Figure S9. The basic information for patients in training group. (A) The gender distribution for the patients. **(B)** The age distribution for each group. The center line in each box represented the median age. **(C)** The number of days between symptom onset and the serum specimen collections at different time points for two different patient groups. Each data point indicates the dpo of a single patient at each time point. The center line in each box shows the median dpo; the bottom and top lines represent the minimal and maximal dpo in each patient group, respectively.

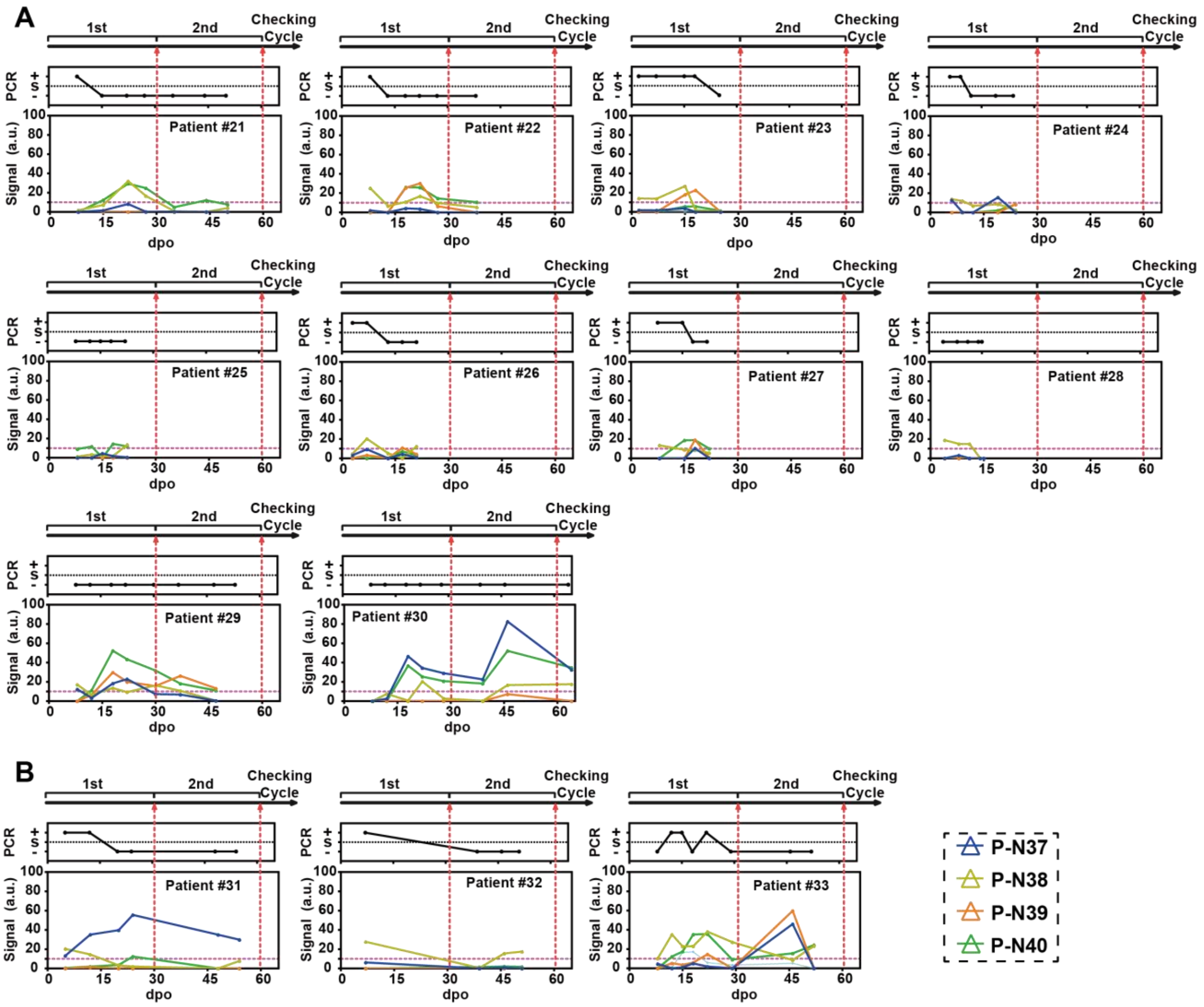

C

	Comparison	"PMI" model					
		1st checking cycle		2nd checking cycle		Summary	
		Moderate-cured	Severe/ Critical	Moderate-cured	Severe/Critical -cured	Moderate-cured	Severe/Critical -cured
Guideline	Moderate-cured (10)	8	2	1	1	9	1
	Severe/Critical -cured (3)	2	1	0	2+1*	0	2+1*

Figure S10. The ECSP IsD curves of P-N37~P-N40 in individual patient from the training group. The PCR result was tracked throughout the disease course for each patient and presented on the top of each panel; while the ECSP IsD curves of P-N37~P-N40 were presented on the bottom of each panel, and the first two checking cycles (1st checking cycle: 0-30 dpo; 2nd checking cycle: 30-60 dpo) were also identified in individual patient. Except for Patient #30, the other patients all passed through the examinations based on the standards of our "PMI" model. (**A**), the patients from the "Moderate-cured" group; (**B**), the

patients from the "Severe/Critical-cured" group. (**C**) Table for comparison of guideline-based vs. PMI Model-based time-resolved diagnosis of disease progression and prognosis. The green number indicates patients which did not require further examinations when using the "PMI" Model; and pink numbers present the one exception compared with guideline (whose real disease progression is in accord with our model); "pink*" presents the one who needs further examinations.

Table S1. Demographic characteristics of the quarantine cohort

	**All**	**Asymptomatic**	**Moderate**	**Severe/critical**	**P value**
	60	18 (30.0%)	33 (55.0%)	9 (15.0%)	
**Age**	44.68±17.23	31.83±15.51	47.30±14.66	60.78±11.30	1.2E-04
**Gender**					
Female	28 (46.7%)	11 (18.3%)	16 (26.7%)	1 (1.7%)	4.7E-02
Male	32 (53.3%)	7 (11.7%)	17 (28.3%)	8 (13.3%)	
**Signs and symptoms**					
Fever	33 (55.0%)	0 (0.0%)	25 (41.7%)	8 (13.3%)	6.6E-01
Fatigue	15 (25.0%)	0 (0.0%)	8 (13.3%)	7 (11.7%)	5.5E-03
Dry cough	24 (40.0%)	0 (0.0%)	17 (28.3%)	7 (11.7%)	2.6E-01
Headache	5 (8.3%)	0 (0.0%)	5 (8.3%)	0 (0.0%)	5.7E-01
Diarrhea	1 (1.7%)	0 (0.0%)	1 (1.7%)	0 (0.0%)	1.0E+00
Pharyngalgia	2 (3.3%)	0 (0.0%)	2 (3.3%)	0 (0.0%)	1.0E+00
Dyspnea	7 (11.7%)	0 (0.0%)	3 (5.0%)	4 (6.7%)	2.8E-02
**Onset of symptom**	5.81±3.07	NA	5.12±2.41	8.33±4.00	2.2E-02
**Hospital admission**	18.07±8.88	9.44±6.05	21.15±6.18	24.00±10.27	3.2E-06
**Corticosteroids**	9 (15.0%)	0 (0.0%)	5 (8.3%)	4 (6.7%)	5.8E-02

"Gender", "Dyspnea", "Onset of symptom": significant difference, p-value < 0.05

"Age", "Fatigue", "Hospital admission": extremely significant difference, p-value < 0.01

The Fisher's exact test or chi-squared test were used to compare qualitative data and the Wilcoxon rank sum test or Kruskal–Wallis H test were used for comparison continuous data.

Table S2. Peptide information of PPHM-1

Peptide	Position	Sequence	Peptide	Position	Sequence
P-S2	11-13	VSSQCVNLTTRTQLPPAYTN	P-S102	1011-1030	QLIRAAEIRASANLAATKMS
P-S3	21-40	RTQLPPAYTNSFTRGVYYPD	P-S103	1021-1040	SANLAATKMSECVLGQSKRV
P-S4	31-50	SFTRGVYYPDKVFRSSVLHS	P-S104	1031-1050	ECVLGQSKRVDFCGKGYHLM
P-S5	41-60	KVFRSSVLHSTQDLFLPFFS	P-S105	1041-1060	DFCGKGYHLMSFPQSAPHGV
P-S6	51-70	TQDLFLPFFSNVTWFHAIHV	P-S106	1051-1070	SFPQSAPHGVVFLHVTYVPA
P-S7	61-80	NVTWFHAIHVSGTNGTKRFD	P-S107	1061-1080	VFLHVTYVPAQEKNFTTAPA
P-S8	71-90	SGTNGTKRFDNPVLPFNDGV	P-S108	1071-1090	QEKNFTTAPAICHDGKAHFP
P-S10	91-110	YFASTEKSNIIRGWIFGTTL	P-S109	1081-1100	ICHDGKAHFPREGVFVSNGT
P-S14	131-150	CEFQFCNDPFLGVYYHKNNK	P-S110	1091-1110	REGVFVSNGTHWFVTQRNFY
P-S15	141-160	LGVYYHKNNKSWMESEFRVY	P-S111	1101-1120	HWFVTQRNFYEPQIITTDNT
P-S18	171-190	VSQPFLMDLEGKQGNFKNLR	P-S114	1131-1150	GIVNNTVYDPLQPELDSFKE
P-S19	181-200	GKQGNFKNLREFVFKNIDGY	P-S115	1141-1160	LQPELDSFKEELDKYFKNHT
P-S20	191-210	EFVFKNIDGYFKIYSKHTPI	P-S116	1151-1170	ELDKYFKNHTSPDVDLGDIS
P-S21	201-220	FKIYSKHTPINLVRDLPQGF	P-S118	1171-1190	GINASVVNIQKEIDRLNEVA
P-S22	211-230	NLVRDLPQGFSALEPLVDLP	P-S119	1181-1200	KEIDRLNEVAKNLNESLIDL
P-S24	231-250	IGINITRFQTLLALHRSYLT	P-S120	1191-1210	KNLNESLIDLQELGKYEQYI
P-S25	241-260	LLALHRSYLTPGDSSSGWTA	P-S121	1201-1220	QELGKYEQYIKWPWYIWLGF
P-S27	261-280	GAAAYYVGYLQPRTFLLKYN	P-S127	1261-1273	SEPVLKGVKLHYT
P-S29	281-300	ENGTITDAVDCALDPLSETK	P-E6	51-70	LVKPSFYVYSRVKNLNSSRV
P-S31	301-320	CTLKSFTVEKGIYQTSNFRV	P-E7	61-75	RVKNLNSSRVPDLLV
P-S32	311-330	GIYQTSNFRVQPTESIVRFP	P-M1	1-20	MADSNGTITVEELKKLLEQW
P-S34	331-350	NITNLCPFGEVFNATRFASV	P-M2	11-30	EELKKLLEQWNLVIGFLFLT
P-S35	341-360	VFNATRFASVYAWNRKRISN	P-M4	31-50	WICLLQFAYANRNRFLYIIK
P-S36	351-370	YAWNRKRISNCVADYSVLYN	P-M11	101-120	RLFARTRSMWSFNPETNILL
P-S37	361-380	CVADYSVLYNSASFSTFKCY	P-M12	111-130	SFNPETNILLNVPLHGTILT
P-S38	371-390	SASFSTFKCYGVSPTKLNDL	P-M14	131-150	RPLLESELVIGAVILRGHLR
P-S39	381-400	GVSPTKLNDLCFTNVYADSF	P-M15	141-160	GAVILRGHLRIAGHHLGRCD
P-S41	401-420	VIRGDEVRQIAPGQTGKIAD	P-M16	151-170	IAGHHLGRCDIKDLPKEITV
P-S42	411-430	APGQTGKIADYNYKLPDDFT	P-M17	161-180	IKDLPKEITVATSRTLSYYK
P-S44	431-450	GCVIAWNSNNLDSKVGGNYN	P-M18	171-190	ATSRTLSYYKLGASQRVAGD
P-S45	441-460	LDSKVGGNYNYLYRLFRKSN	P-M19	181-200	LGASQRVAGDSGFAAYSRYR
P-S46	451-470	YLYRLFRKSNLKPFERDIST	P-M20	191-210	SGFAAYSRYRIGNYKLNTDH
P-S48	471-490	EIYQAGSTPCNGVEGFNCYF	P-M21	201-220	IGNYKLNTDHSSSSDNIALL
P-S49	481-500	NGVEGFNCYFPLQSYGFQPT	P-N2	11-30	NAPRITFGGPSDSTGSNQNG
P-S50	491-510	PLQSYGFQPTNGVGYQPYRV	P-N3	21-40	SDSTGSNQNGERSGARSKQR
P-S51	501-520	NGVGYQPYRVVVLSFELLHA	P-N5	41-60	RPQGLPNNTASWFTALTQHG
P-S52	511-530	VVLSFELLHAPATVCGPKKS	P-N6	51-70	SWFTALTQHGKEDLKFPRGQ
P-S53	521-540	PATVCGPKKSTNLVKNKCVN	P-N7	61-80	KEDLKFPRGQGVPINTNSSP
P-S54	531-550	TNLVKNKCVNFNFNGLTGTG	P-N8	71-90	GVPINTNSSPDDQIGYYRRA
P-S55	541-560	FNFNGLTGTGVLTESNKKFL	P-N10	91-110	TRRIRGGDGKMKDLSPRWYF
P-S56	551-570	VLTESNKKFLPFQQFGRDIA	P-N11	101-120	MKDLSPRWYFYYLGTGPEAG
P-S57	561-580	PFQQFGRDIADTTDAVRDPQ	P-N12	111-130	YYLGTGPEAGLPYGANKDGI
P-S58	571-590	DTTDAVRDPQTLEILDITPC	P-N13	121-140	LPYGANKDGIIWVATEGALN
P-S61	601-620	GTNTSNQVAVLYQDVNCTEV	P-N14	131-150	IWVATEGALNTPKDHIGTRN
P-S62	611-630	LYQDVNCTEVPVAIHADQLT	P-N15	141-160	TPKDHIGTRNPANNAAIVLQ
P-S63	621-640	PVAIHADQLTPTWRVYSTGS	P-N16	151-170	PANNAAIVLQLPQGTTLPKG
P-S64	631-650	PTWRVYSTGSNVFQTRAGCL	P-N17	161-180	LPQGTTLPKGFYAEGSRGGS
P-S66	651-670	IGAEHVNNSYECDIPIGAGI	P-N19	181-200	QASSRSSSRSRNSSRNSTPG
P-S68	671-690	CASYQTQTNSPRRARSVASQ	P-N20	191-210	RNSSRNSTPGSSRGTSPARM
P-S69	681-700	PRRARSVASQSIIAYTMSLG	P-N21	201-220	SSRGTSPARMAGNGGDAALA
P-S76	751-770	NLLLQYGSFCTQLNRALTGI	P-N22	211-230	AGNGGDAALALLLLDRLNQL
P-S78	771-790	AVEQDKNTQEVFAQVKQIYK	P-N23	221-240	LLLLDRLNQLESKMSGKGQQ
P-S79	781-800	VFAQVKQIYKTPPIKDFGGF	P-N24	231-250	ESKMSGKGQQQQGQTVTKKS
P-S80	791-810	TPPIKDFGGFNFSQILPDPS	P-N25	241-260	QQGQTVTKKSAAEASKKPRQ
P-S82	811-830	KPSKRSFIEDLLFNKVTLAD	P-N26	251-270	AAEASKKPRQKRTATKAYNV
P-S83	821-840	LLFNKVTLADAGFIKQYGDC	P-N27	261-280	KRTATKAYNVTQAFGRRGPE
P-S84	831-850	AGFIKQYGDCLGDIAARDLI	P-N28	271-290	TQAFGRRGPEQTQGNFGDQE
P-S85	841-860	LGDIAARDLICAQKFNGLTV	P-N29	281-300	QTQGNFGDQELIRQGTDYKH
P-S86	851-870	CAQKFNGLTVLPPLLTDEMI	P-N30	291-310	LIRQGTDYKHWPQIAQFAPS
P-S90	891-910	GAALQIPFAMQMAYRFNGIG	P-N31	301-320	WPQIAQFAPSASAFFGMSRI
P-S91	901-920	QMAYRFNGIGVTQNVLYENQ	P-N34	331-350	LTYTGAIKLDDKDPNFKDQV
P-S92	911-930	VTQNVLYENQKLIANQFNSA	P-N35	341-360	DKDPNFKDQVILLNKHIDAY
P-S93	921-940	KLIANQFNSAIGKIQDSLSS	P-N36	351-370	ILLNKHIDAYKTFPPTEPKK
P-S94	931-950	IGKIQDSLSSTASALGKLQD	P-N37	361-380	KTFPPTEPKKDKKKKADETQ
P-S95	941-960	TASALGKLQDVVNQNAQALN	P-N38	371-390	DKKKKADETQALPQRQKKQQ
P-S96	951-970	VVNQNAQALNTLVKQLSSNF	P-N39	381-400	ALPQRQKKQQTVTLLPAADL
P-S98	971-990	GAISSVLNDILSRLDKVEAE	P-N40	391-410	TVTLLPAADLDDFSKQLQQS
P-S101	1001-1020	LQSLQTYVTQQLIRAAEIRA	P-N41	401-419	DDFSKQLQQSMSSADSTQA

Table S3. Peptide information of PPHM-2 (partial peptides from PPHM-1)

Peptide	Position	Sequence
P-S4	31-50	SFTRGVYYPDKVFRSSVLHS
P-S7	61-80	NVTWFHAIHVSGTNGTKRFD
P-S15	141-160	LGVYYHKNNKSWMESEFRVY
P-S21	201-220	FKIYSKHTPINLVRDLPQGF
P-S31	301-320	CTLKSFTVEKGIYQTSNFRV
P-S37	361-380	CVADYSVLYNSASFSTFKCY
P-S39	381-400	GVSPTKLNDLCFTNVYADSF
P-S44	431-450	GCVIAWNSNNLDSKVGGNYN
P-S52	511-530	VVLSFELLHAPATVCGPKKS
P-S53	521-540	PATVCGPKKSTNLVKNKCVN
P-S56	551-570	VLTESNKKFLPFQQFGRDIA
P-S57	561-580	PFQQFGRDIADTTDAVRDPQ
P-S58	571-590	DTTDAVRDPQTLEILDITPC
P-S63	621-640	PVAIHADQLTPTWRVYSTGS
P-S64	631-650	PTWRVYSTGSNVFQTRAGCL
P-S69	681-700	PRRARSVASQSIIAYTMSLG
P-S78	771-790	AVEQDKNTQEVFAQVKQIYK
P-S82	811-830	KPSKRSFIEDLLFNKVTLAD
P-S83	821-840	LLFNKVTLADAGFIKQYGDC
P-S95	941-960	TASALGKLQDVVNQNAQALN
P-S102	1011-1030	QLIRAAEIRASANLAATKMS
P-S104	1031-1050	ECVLGQSKRVDFCGKGYHLM
P-S108	1071-1090	QEKNFTTAPAICHDGKAHFP
P-S111	1101-1120	HWFVTQRNFYEPQIITTDNT
P-S115	1141-1160	LQPELDSFKEELDKYFKNHT
P-S118	1171-1190	GINASVVNIQKEIDRLNEVA
P-E6	51-70	LVKPSFYVYSRVKNLNSSRV
P-M1	43850	MADSNGTITVEELKKLLEQW
P-M15	141-160	GAVILRGHLRIAGHHLGRCD
P-M16	151-170	IAGHHLGRCDIKDLPKEITV
P-M17	161-180	IKDLPKEITVATSRTLSYYK
P-N15	141-160	TPKDHIGTRNPANNAAIVLQ
P-N16	151-170	PANNAAIVLQLPQGTTLPKG
P-N17	161-180	LPQGTTLPKGFYAEGSRGGS
P-N22	211-230	AGNGGDAALALLLLDRLNQL
P-N23	221-240	LLLLDRLNQLESKMSGKGQQ
P-N24	231-250	ESKMSGKGQQQQGQTVTKKS
P-N25	241-260	QQGQTVTKKSAAEASKKPRQ
P-N26	251-270	AAEASKKPRQKRTATKAYNV
P-N36	351-370	ILLNKHIDAYKTFPPTEPKK
P-N37	361-380	KTFPPTEPKKDKKKKADETQ
P-N38	371-390	DKKKKADETQALPQRQKKQQ
P-N39	381-400	ALPQRQKKQQTVTLLPAADL
P-N40	391-410	TVTLLPAADLDDFSKQLQQS
P-N41	401-419	DDFSKQLQQSMSSADSTQA
